# P-1404. Optimizing Preclinical Models and Therapeutic Combinations for Nontuberculous Mycobacterial Lung Disease

**DOI:** 10.1093/ofid/ofaf695.1591

**Published:** 2026-01-11

**Authors:** Ruth A Howe, Chandra M Panthi, Binayak Rimal, Gyanu Lamichhane

**Affiliations:** Johns Hopkins Medicine, Baltimore, MD; Johns Hopkins Medicine, Baltimore, MD; Johns Hopkins Medicine, Baltimore, MD; Johns Hopkins University/School of Medicine, Baltimore, Maryland

## Abstract

**Background:**

Pulmonary disease caused by the nontuberculous mycobacteria (NTMs) *Mycobacteroides abscessus* (*Mab*) and *M. avium* (*MAC*) is a rising clinical concern, with increased incidence above expectation from advances in detection. Prolonged, multimodal antibiotic treatment that often relies on a parenteral agent remains burdensome for patients with low cure rates and high morbidity. More tolerable and effective therapeutic combinations are needed, with a focus on orally bioavailable agents. This in turn requires more accurate preclinical modeling than currently possible with the single available *Mab* reference strain.

**Methods:**

For *Mab* preclinical model testing, mice were exposed to one of 16 clinical pulmonary *Mab* isolates or the reference strain ATCC 19977 via aerosol and followed with lungs, spleen, and liver collected every 2 weeks. Disease burden was assessed by colony forming unit assay and by histopathologic analysis. For drug synergy testing, combinations were first validated *in vitro* by full checkerboard assay. For *in vivo* studies, NTM-infected mice were administered either placebo, single-agent, dual agent, or triple agent regimens. Pulmonary disease burden was assessed at 2-week intervals alongside serial drug MIC testing on recovered organisms.

**Results:**

ATCC 19977 resulted in early, aggressive disease dissemination and less pulmonary disease burden than the majority of clinical pulmonary *Mab* isolates. Four clinical isolates, one each for smooth and rough phenotypes of the subspecies *massiliense* and *abscessus*, were identified with high lung disease penetration and delayed disease dissemination. Multiple dual-agent combinatorial treatments reduced pulmonary NTM burden comparably to standard of care in *in vivo* murine pulmonary NTM infection.

**Conclusion:**

Here we present four improved murine models for *Mab* pulmonary disease for both clinically relevant subspecies with higher lung disease burden and reduced systemic dissemination compared to the current reference strain. This will permit more accurate modeling and drug testing for multiple *Mab* pulmonary disease scenarios with reduced preclinical overhead. We further find synergy between multiple orally bioavailable agents that may permit simplified, more tolerable regimens early in NTM pulmonary disease treatment.

**Disclosures:**

All Authors: No reported disclosures

